# Residual Viremia in an RT-SHIV Rhesus Macaque HAART Model Marked by the Presence of a Predominant Plasma Clone and a Lack of Viral Evolution

**DOI:** 10.1371/journal.pone.0088258

**Published:** 2014-02-05

**Authors:** Robert C. Kauffman, Andradi Villalobos, Joanne H. Bowen, Lourdes Adamson, Raymond F. Schinazi

**Affiliations:** 1 Center for Comparative Medicine, University of California Davis, Davis, California, United States of America; 2 Department of Pediatrics, Laboratory of Biochemical Pharmacology, Center for AIDS Research, Emory University School of Medicine, Atlanta, Georgia, United States of America; 3 Veterans Affairs Medical Center, Decatur, Georgia, United States of America; Centro de Biología Molecular Severo Ochoa (CSIC-UAM), Spain

## Abstract

Highly active antiretroviral therapy (HAART) significantly reduces HIV-1 replication and prevents progression to AIDS. However, residual low-level viremia (LLV) persists and long-lived viral reservoirs are maintained in anatomical sites. These reservoirs permit a recrudescence of viremia upon cessation of therapy and thus HAART must be maintained indefinitely. HIV-1 reservoirs include latently infected resting memory CD4^+^ T-cells and macrophages which may contribute to residual viremia. It has not been conclusively determined if a component of LLV may also be due to residual replication in cells with sub-therapeutic drug levels and/or long-lived chronically infected cells. In this study, RT-SHIV_mac239_ diversity was characterized in five rhesus macaques that received a five-drug HAART regimen [tenofovir, emtricitabine, zidovudine, amdoxovir, (A, C, T, G nucleoside analogs) and the non-nucleoside reverse transcriptase (RT) inhibitor efavirenz]. Before maximal viral load suppression, longitudinal plasma viral RNA RT diversity was analyzed using a 454 sequencer. After suppression, LLV RT diversity (amino acids 65-210) was also assessed. LLV samples had viral levels less than our standard detection limit (50 viral RNA copies/mL) and few transient blips <200 RNA copies/mL. HAART was discontinued in three macaques after 42 weeks of therapy resulting in viral rebound. The level of viral divergence and the prevalence of specific alleles in LLV was similar to pre-suppression viremia. While some LLV sequences contained mutations not observed in the pre-suppression profile, LLV was not characterized by temporal viral evolution or apparent selection of drug resistance mutations. Similarly, resistance mutations were not detected in the viral rebound population. Interestingly, one macaque maintained a putative LLV predominant plasma clone sequence. Together, these results suggest that residual replication did not markedly contribute to LLV and that this model mimics the prevalence and phylogenetic characteristics of LLV during human HAART. Therefore, this model may be ideal for testing HIV-1 eradication strategies.

## Introduction

Highly active antiretroviral therapy (HAART) delays progression to acquired immunodeficiency syndrome (AIDS) in most individuals infected with the human immunodeficiency virus type 1 (HIV-1). HAART is characterized by the use of combination drug therapy that consists of three or more antiretroviral drugs from at least two different classes [1]. Once individuals begin HAART, a rapid reduction in plasma viral RNA (vRNA) occurs and the plasma viral load (VL) is frequently suppressed to levels below the detection sensitivity of standard assays (<50 vRNA copies/mL) [2]–[5]. Despite VL suppression, transient blips >50 vRNA copies/mL occur [6]; however, in compliant individuals, blips rarely signify the development of drug resistance [7]. More sensitive VL assays with single vRNA copy sensitivity have demonstrated that in most individuals, residual low-level viremia (LLV) is present during HAART [2], [5] at levels <50 vRNA copies/mL. In addition, cessation of therapy results in a rapid rebound in viremia presumably from sites of long-term viral persistence that are not eliminated by HAART [7]–[11]. Viral persistence has been well documented in long-lived viral reservoirs consisting of latently infected resting memory CD4^+^ T-cells [12]–[15]. Moreover, infected macrophages (reviewed in [16]) may also represent an important long-lived reservoir that can produce virus throughout their life span due to the fact that these cells are resistant to viral cytopathic effects.

It has been suggested that virions within LLV [17], [18] and rebound viremia [19] are often not the exclusive result of viral production from circulating resting memory CD4^+^ T-cells. This suggests that viral reservoirs within tissues may be a primary source of LLV and rebound viremia, however, the origin of these virions has yet to be fully characterized. HIV-1 infected cells within these anatomical sites may contribute to LLV following cellular activation or transient viral production. Additionally, it has yet to be conclusively determined if a component of LLV may also be due to instances of complete replication cycles, termed residual replication, in cells with sub-therapeutic drug levels [20]. The ability to definitively ascertain the occurrence of residual replication during HAART is the primary goal of many current research efforts and may contribute to improved strategies toward HIV-1 eradication [21] and control of chronic immune activation (reviewed in [22]). Using phylogenetic analyses of LLV, studies have reported that residual replication may occur in some patients [23], [24] while others have found no substantial evidence of ongoing HIV-1 replication during HAART [17], [18], [25]. Phylogenetic studies of human LLV have also demonstrated that it is frequently marked by the existence of an oligoclonal population of viral sequences deemed predominant plasma clones (PPC) [17]. A linkage between PPC viral populations and viral nucleic acid sequences in circulating resting memory CD4^+^ T-cells has not been well defined in the majority of studies that have reported this phenomenon [17], [18], [25]–[27]. One study performed by Anderson *et al.* reported that a minority of circulating resting memory CD4^+^ T-cells did harbor PPC sequences suggesting that they may have arisen from homeostatic proliferation [25]. Taken together, the mechanisms contributing to the production of PPC viral populations are largely unknown. In addition to residual replication contributing to LLV, it has been proposed that the rapid rebound in viremia, which occurs upon cessation of therapy, may originate from virions present due to residual replication or persistent low-level viral production [26], [28], [29]. However, in the majority of individuals, activation of the latent viral reservoir has been implicated as the primary cause of rebound viremia [28], [30].

To investigate questions regarding viral persistence, residual replication, and LLV during therapy, non-human primate models of HIV-1 HAART have been developed [31]–[35]. The use of a non-human primate model that mimics the viral dynamics of HIV-1 infected humans during HAART has several advantages. These include improved compliance, the use of a genetically defined inoculum, and the ability to perform invasive tissue sampling. Recently, Shytaj *et al*. monitored LLV VLs for 14 to 94 weeks during HAART following an intensified HAART regimen in SIV_mac251_ infected rhesus macaques (RMs) [35]. However, in nine of the ten RMs, LLV was completely suppressed to undetectable levels (<3 vRNA copies/mL) suggesting that this model may have limitations in modeling human residual viremia. Other studies have used RT-SHIV [31], [32], [34] which is a chimeric simian immunodeficiency virus (SIV) with the HIV-1 reverse transcriptase (RT) replacing the native SIV RT [36]. One benefit of RT-SHIV is that unlike SIV, the chimeric virus is susceptible to non-nucleoside RT inhibitors (NNRTIs) [37] which are an important component of frontline treatment regimens [38], [39]. Similar to humans [4], plasma VLs in RT-SHIV infected RMs decay in a bi-phasic manner and are rapidly suppressed to <50 vRNA copies/mL within the first three months of HAART [32], [40]. Viral loads are maintained at this level of suppression for the duration of therapy and are associated with few transient blips in viremia (<200 vRNA copies/mL) at frequencies comparable to humans [6]. In addition, our group has demonstrated that RT-SHIV infected RMs which received a three-drug HAART regimen maintained persistent LLV for at least 20 weeks following VL suppression to levels <50 vRNA copies/mL [41]. Moreover, LLV vRNA levels [41] were comparable to those observed in humans during the first year of therapy [4], [5]. The specific anatomical origins of this LLV were not determined. However, in tissues collected at necropsy during maximal VL suppression (VL: <50 vRNA copies/mL), RT-SHIV cell-associated viral DNA and full-length vRNA were present in many anatomical sites such as lymphoid tissues and the gastrointestinal tract [32].

In this study, our primary goals were to characterize LLV genetic diversity in a non-human primate model of HIV-1 HAART and to investigate the controversial hypothesis that residual replication occurs and may contribute to LLV. To investigate these questions, we analyzed the genetic diversity of RT-SHIV RT sequences in longitudinally sampled plasma that was obtained before maximal VL suppression and during maximally suppressive therapy. Plasma samples were kindly provided from a concurrent study which was designed, in part, to assess the hypothesis that a five-drug HAART regimen [tenofovir, emtricitabine, zidovudine, amdoxovir, (A, C, T, G nucleoside analogs) and the NNRTI efavirenz] may differentially affect LLV and viral rebound relative to a more traditional three-drug HAART regimen. Only one other similar non-human primate study has been performed to date which was reported in 2011 by Kearney *et al*. [31]. During that important study, the ability to achieve complete VL suppression in all six RT-SHIV infected pigtailed macaques was likely affected by the development of drug resistance following an initial period of efavirenz monotherapy in some macaques. The reported phylogenetic diversity of LLV samples with <50 vRNA copies/mL was limited to two pigtailed macaques for a period of five weeks [31]. In these samples, longitudinal viral evolution was not detected and viral diversity was similar to the plasma viral RNA pre-suppression profile [31]. These findings suggest that residual replication does not markedly contribute to LLV in well suppressed pigtailed macaques. Conversely, evidence of viral evolution during HAART was detected in viremia from four pigtailed macaques that did not achieve maximal VL suppression [31]. In comparison, this study attempted to improve the characterization of LLV by analyzing samples from five rhesus macaques that all achieved maximal VL suppression (<50 vRNA copies/mL). In addition, we were able to generate LLV sequences from longer periods ranging from 10 to 32 weeks. The results of these analyses are reported herein.

## Results

### Longitudinal analysis of viremia

**Figure 1:**
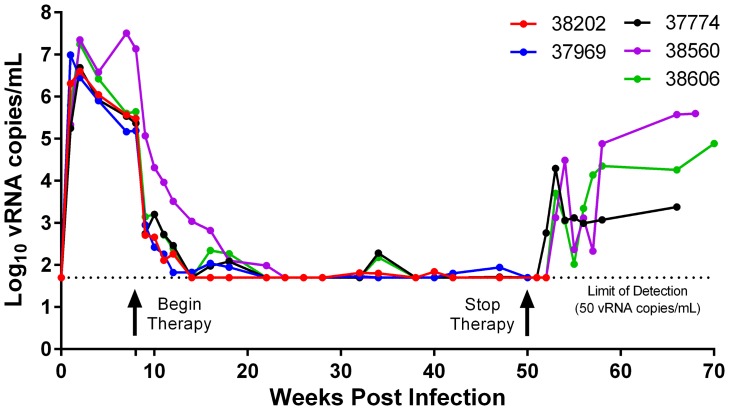
Longitudinal analysis of plasma viral loads. Plasma viral loads were determined by TaqMan RT-qPCR using our standard viral load assay. All RMs began HAART (4 NRTI, 1 NNRTI) after eight weeks of infection. Rhesus macaques Mmu 37969 and Mmu 38202 were necropsied during therapy at weeks 50 and 52 PI respectively. Viremia rebounded in RMs Mmu 38560, Mmu 38606, and Mmu 37774 upon cessation of therapy at week 50 PI. These RMs were necropsied on the following weeks PI: (Mmu 37774: week 65, Mmu 38560: week 67, and Mmu 38606: week 69). The dotted line indicates the lower limit of detection of the standard viral load assay (50 copies of viral RNA per mL of plasma).

Five female RMs were infected with RT-SHIV after a single intravenous inoculation. Peak viremia was achieved within two weeks post infection (PI) and ranged from 4×10^6^ to 2.2×10^7^ vRNA copies/mL (Figure 1). HAART was initiated eight weeks PI and, within two weeks of treatment, the plasma VL decreased by an average of 2.4 log_10_ vRNA copies/mL (95% Confidence Interval (CI), 2.1 to 2.8). After initiation of therapy, viremia decreased to <50 vRNA copies/mL in all five RMs; however, the time to achieve maximal VL suppression was highly variable (6 to 16 weeks post-HAART). In general, viremia was maintained at levels less than 50 vRNA copies/mL during HAART; nevertheless, rare transient blips in LLV that were between 50 and 200 vRNA copies/mL were observed in four RMs (Figure 1; Mmu 37774, Mmu 37969, Mmu 38606, and Mmu 38202). The occurrence of VL blips did not directly correspond to a reduction in the dose of any antiretroviral drugs. Both RMs Mmu 37969 and Mmu 38202 were necropsied during therapy at weeks 50 and 52 PI respectively and had VLs <50 vRNA copies/mL. Therapy was discontinued in the remaining three RMs at week 50 PI, resulting in a rapid rebound in viremia (Figure 1). Viremia was first observed in RM Mmu 37774 at week 52 PI (VL: 5.8×10^2^ vRNA copies/mL) and peaked at week 53 PI (VL: 2.0×10^4^ vRNA copies/mL). Initial viral rebound occurred at week 53 PI for RM Mmu 38560 (VL: 1.3×10^3^ vRNA copies/mL and peaked at week 54 PI (VL: 3.0×10^4^ vRNA copies/mL). Finally, in RM Mmu 38606, both initial and peak viral rebound occurred at week 53 PI (VL: 5.0×10^3^ vRNA copies/mL).

### Generation of the pre-suppression profile and low-level viremia sequences

In this study, vRNA within plasma obtained before maximal VL suppression (<50 vRNA copies/mL) was sequenced in order to characterize the “pre-suppression profile” of RT-SHIV mutations and viral divergence. Specifically, the pre-suppression profile was generated from plasma sampled at weeks 2, 4, and 8 PI and, in general, every two weeks after the initiation of HAART (week 8 PI) until VLs were suppressed to levels less than 200 vRNA copies/mL. The time to achieve this level of VL suppression was different for each RM (Figure 1), and thus the pre-suppression profile of each RM represents a variable number of time points between weeks 2 and 18 PI (Table 1). Using these samples, we sequenced the region of RT encoding amino acids 41 to 296. The targeted region was sequenced in two overlapping amplicons using a 454 sequencer (454-RT amplicon 1: amino acids 41 to 175 and 454-RT amplicon 2: amino acids 157 to 296; Tables S1 and S2). Deep sequencing of PCR amplicons generated an average of 1,288 reads/amplicon (95% CI, 1143 to 1432) (Sequences S1). In order to assess the level of divergence in LLV, partial RT sequences encoding amino acids 65-210 were generated by single-genome amplification (SGA) from plasma samples collected only after maximal VL suppression had been achieved (<50 vRNA copies/mL). Preliminary analyses of LLV samples indicated that complete RT sequences could not be efficiently generated with the available methodology. This may have been influenced by low VLs and a limited availability of target molecules. The sampling protocol used in this study permitted a single 4–5 mL plasma sample collection every two weeks. Therefore, in order to improve the success rate of generating LLV sequences, the amplicon size was intentionally constrained.

**Table 1:** LLV mutations and their abundance in the pre-suppression profile^a^

Macaque ID Mmu:	LLV RT mutations			Pre-suppression profile: Weeks post infection							
; (# LLV Sequences)	Mutation (nt)	Amino Acid	#^b^	2	4	8	10	12	14	16	18
38202; (27)	A198G	K66 =	1	0.6	1.0	c	9.1	ND	ND	ND	ND
	T402C	S134 =	1	-	-	c	-	ND	ND	ND	ND
	C540T	I180 =	3	-	-	-	-	ND	ND	ND	ND
	G586A	G196R	17	43	56	80	86	ND	ND	ND	ND
	G612A	E204 =	1	-	0.8	0.6	-	ND	ND	ND	ND
	A623T	H208L	1	-	1.7	-	18	ND	ND	ND	ND
37969; (15)	G364A	E122K	8	-	-	0.8	40^d^	ND	ND	ND	ND
	G586A	G196R	6	34	60	70	40^d^	ND	ND	ND	ND
	G612A	E204 =	8	-	-	0.6	20^d^	ND	ND	ND	ND
	PPC^e^	PPC^e^	8	-	-	-	40^d^	ND	ND	ND	ND
38606; (9)	A200G	D67G	1	5.2	13	4.2	7.9	-	ND	ND	ND
	G263A	W88(*)	1	-	-	-	-	-	ND	ND	ND
	*G412A*	*E138K*	1	-	-	-	-	-	ND	ND	ND
	G428A	R143K	1	-	-	-	-	-	ND	ND	ND
	*G485A*	*S162N*	1	-	-	1.7	1.4	-	ND	ND	ND
	A490G	M164V	1	-	-	-	-	-	ND	ND	ND
	*A521G*	*Q174R*	1	0.7	0.7	1.0	-	-	ND	ND	ND
	G586A	G196R	5	47	63	72	73	c	ND	ND	ND
37774; (3)	G586A	G196R	3	46	69	96	76	ND	ND	ND	ND
38560; (9)	G586A	G196R	3	43	7.7	1.3	c	ND	-	-	17
	A623T	H208L	9	5.4	88	100	c	ND	86	88	89

a Mutations are numbered beginning with the first nucleotide/amino acid of RT. The region encoding RT amino acids 65-210 was sequenced from LLV samples. Mutation abundance in the pre-suppression profile is expressed as the percentage of 454 sequence reads encoding the given mutation. Dashes (-) indicate that the specified mutation was not observed in sequence reads at a pre-determined 0.5% read threshold. LLV mutations that were not observed in the pre-suppression profile are underlined. LLV mutations associated with RT drug resistance are indicated by bold italics. ND, not determined: samples were either unavailable or not tested because viral loads were below the amplification sensitivity of the 454 sequencing assay. Nucleotide: (nt)

b Number of LLV sequences containing a specific mutation (shown at left).

c Sample was processed; however, sequence data was not generated at this position.

d Sample analyzed by single-genome amplification (SGA) and not 454 sequencing. Five sequences were generated by SGA at week 10 PI for RM Mmu 37969.

e The LLV predominant plasma clone (PPC) mutation in RM Mmu 37969 was characterized by the following ten linked RT nucleotide substitutions: C258T, C291T, A304C,T336C, G364A, A378G, A483G, A484T, T537C, and G612A. Nine of these mutations were linked on two individual sequences obtained at week 10 PI. G612A was only present in one of the week 10 PI sequences and is listed separately.

Virions within pre-suppression viremia were first concentrated by ultracentrifugation before vRNA extraction. After extraction, qRT-PCR analysis of vRNA determined that VLs were equivalent to those measured by our standard assay (data not shown). This indicates that the ultracentrifugation procedure enhanced population sampling due to the fact that 20 to 35 times more plasma was used for vRNA extraction versus our standard plasma vRNA extraction protocol. The majority of amplicons generated to characterize the pre-suppression profile originated from cDNA molecules derived from vRNA templates; however, qRT-PCR analysis demonstrated that low-level viral DNA (vDNA) contamination existed in vRNA samples. In these samples, the concentration of vDNA was ≤0.1% of the vRNA concentration (data not shown). Following maximal VL suppression, 65% of the assayed plasma samples generated at least one LLV sequence by SGA (Table 2). However, marked inter-RM variability in the ability to generate LLV sequences was observed. This is highlighted by RM Mmu 37774 from which only two early plasma samples (weeks 18 and 28 PI) yielded a combined total of three sequences and later time points were PCR negative for LLV sequences (Table 2). In order to estimate the level of LLV vDNA contamination after sample processing, a total of ten samples with VLs <50 vRNA copies/mL were tested by qRT-PCR. These samples were derived from three different RMs and included samples from RM Mmu 38471 which was experimentally identical to the other five RMs but was not used for sequence analyses. Of these ten samples, eight had detectable viral nucleic acids in reactions containing Accuscript reverse transcriptase and only one sample had detectable vDNA (10% of total vRNA/vDNA) in control reactions lacking reverse transcriptase (data not shown). Despite these encouraging findings, there is an inherent level of uncertainty that all LLV sequences reported in this study were derived from a cDNA template.

**Table 2:** Sample viral load and number of LLV sequences/sample^a^

	Rhesus Macaque ID Mmu:				
Weeks PI	38202 (VL^b^)	37969 (VL^b^)	38606 (VL^b^)	37774 (VL^b^)	38560 (VL^b^)
**18**	5 (<50)^c^	7 (90)		2 (120)	
**22**	3 (<50)				
**26**	8 (<50)				
**28**	0 (<50)	0 (<50)		1 (<50)	
**30**	4 (<50)^c^				
**34**	0 (60)	2 (<50)	2 (150)	0 (190)	2 (<50)
**38**	5 (<50)	0 (<50)	4 (<50)^c^	0 (<50)	5 (<50)
**45**	1 (<50)	1 (<50)	0 (<50)	0 (<50)	1 (<50)
**47**	0 (<50)	2 (90)			
**48**			3 (<50)^c^	0 (<50)	1 (<50)
**50**	0 (<50)	4^d^ (<50), (7)^e^			
**52**	0^d^(<50), (<2)^e^				

a The number of sequences generated in each plasma sample is shown to the left of the sample's viral load which is denoted in parentheses.

b Viral load (VL) was determined by our standard viral load assay which has a sensitivity of 50 vRNA copies/mL.

c Time points where some LLV sequences contained mutations which were not observed in the pre-suppression profile

d Samples were obtained at necropsy and approximately 20 mL of plasma was analyzed to generate LLV sequences.

e Viral load determined by an unmodified ultracentrifugation viral load assay (Deere *et al.* [48] using triplicate 10 µL vRNA samples. Sensitivity was 2 vRNA copies/mL.

### Analysis of sequencing errors and assay sensitivity

The error rate of the Phusion high-fidelity DNA polymerase was estimated by using SGA to amplify a 1.8 kb region of RT from the 5′ half clone of RT-SHIV. A total of 29 amplicons were sequenced. Three separate sequences each contained a single ambiguous base (two G-to-T and one C-to-A), indicating a substitution error rate of 0.006%. This estimated error rate closely agrees with the 0.01% Phusion substitution error rate reported by Vandenbroucke *et al*. [42]. Of the seven high fidelity DNA polymerases measured in their study, Phusion had the lowest substitution error rate [42]. In order to generate sequenceable PCR amplicons, a high-fidelity reverse transcriptase (Accuscript) was used to generate template cDNA molecules from vRNA. However, the substitution error rate of AccuScript RT was not directly measured and it cannot be excluded that some mutations reported in this study may have been introduced during cDNA synthesis. The error rate of PCR through 454-sequencing was estimated by sequencing the gp120 region of envelope using 1×10^4^ copies of the 3′ RT-SHIV plasmid half clone. Approximately 7,000 reads were generated per base, and only one nucleotide substitution (Env-C1128A) was present in both read orientations in 0.51% of reads (data not shown). When this experiment was repeated, the same mutation was identified again in 0.46% of reads. This may indicate that this mutation was a natural polymorphism in the plasmid stock and not introduced experimentally. Collectively, it was determined that, after data filtration, a sequencing read threshold value of 0.5% was adequate to exclude most PCR and sequencing errors.

In order to estimate the mutation detection sensitivity, two necropsy derived plasma samples were processed in parallel from RM Mmu 38560. This RM had the highest necropsy VL (3.9×10^5^ vRNA copies/mL) (Figure 1). We chose to sequence the region encoding RT amino acids 41 to 296 and all of envelope gp120 using a 454 sequencer. Approximately 1,500 reads per base were generated. This sequencing depth is comparable to the pre-suppression profile (Sequences S1). This analysis detected 96% (28 of 29) of mutations >2% of reads that were in each sample; however, these two samples only shared 38% (19 of 50) of the observed mutations <2% of reads (data not shown). We did not determine if limitations in the mutation detection sensitivity of the 454 sequencing assay could be fully overcome by either markedly increasing the sequencing depth or analyzing a single amplicon. Nevertheless, an increased investment in sequencing may not be able to detect all minor viral mutations within a diverse plasma population given the strict sampling limitations in both non-human primate and human research.

### Comparison of low-level viremia divergence and allele frequency to pre-suppression viremia

**Figure 2:**
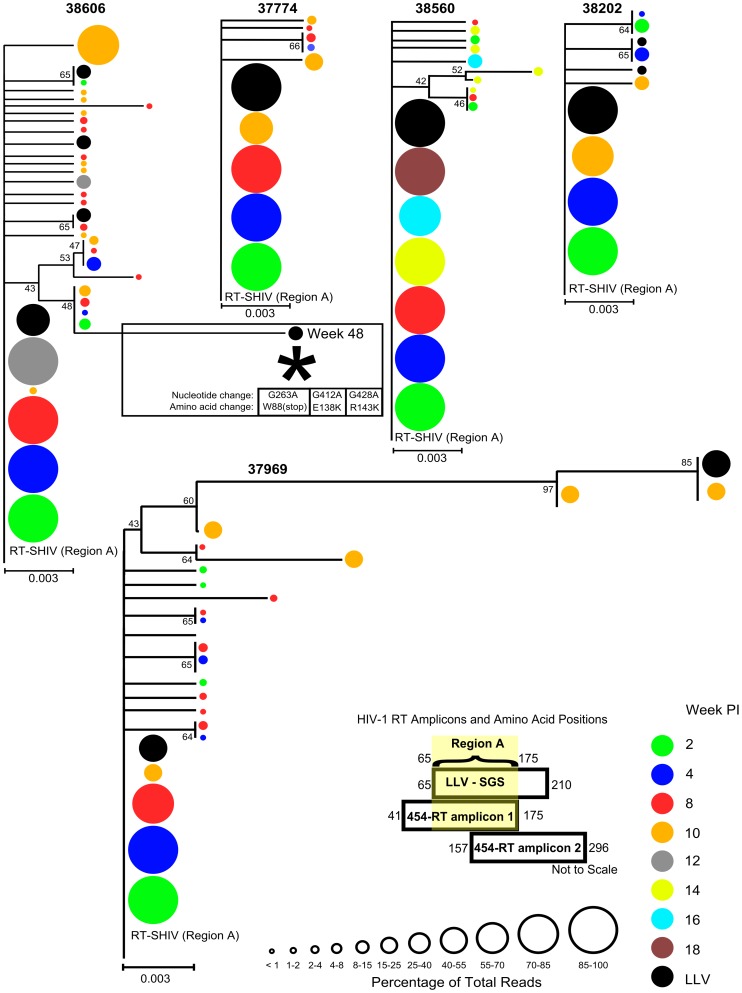
Comparative phylogenetic analysis of pre-suppression and low-level viremia (RT amino acids 65–175). Multiple sequence alignments were performed using 454-RT amplicon 1 pre-suppression 454 consensus sequences and SGA LLV sequences. There were a total of 333 nucleotide positions in the alignment encoding RT amino acids 65 to 175 (region A). The evolutionary history was inferred using the Neighbor-Joining method [75]. The percentage of replicate trees in which the associated taxa clustered together in the bootstrap test (1000 replicates) are shown next to the branches [78]. Only values greater than 40 have been shown. The tree is drawn to scale, with branch lengths in the same units as those of the evolutionary distances used to infer the phylogenetic tree. The evolutionary distances were computed using the Tamura-Nei method [76] and are in the units of the number of base substitutions per site. All positions containing gaps and missing data were eliminated. All phylograms were rooted on the consequence sequence of the RT-SHIV inoculum. Evolutionary analyses were conducted in MEGA5 [73]. The number of reads at each node were binned based upon the percentage of 454 sequence reads containing the indicated sequence at each time point. The binning scale is shown by progressively sized open circles. The week post infection (PI) from which pre-suppression sequences were derived is distinguished by different colors. All LLV sequences were obtained following maximal viral load suppression and are grouped together (black circles). The asterisk denoting the Mmu 38606 week 48 LLV sequence indicates that this sequence was more divergent than the maximal level of divergence in the pre-suppression profile (0.012 vs 0.006 mutations/site).

**Figure 3:**
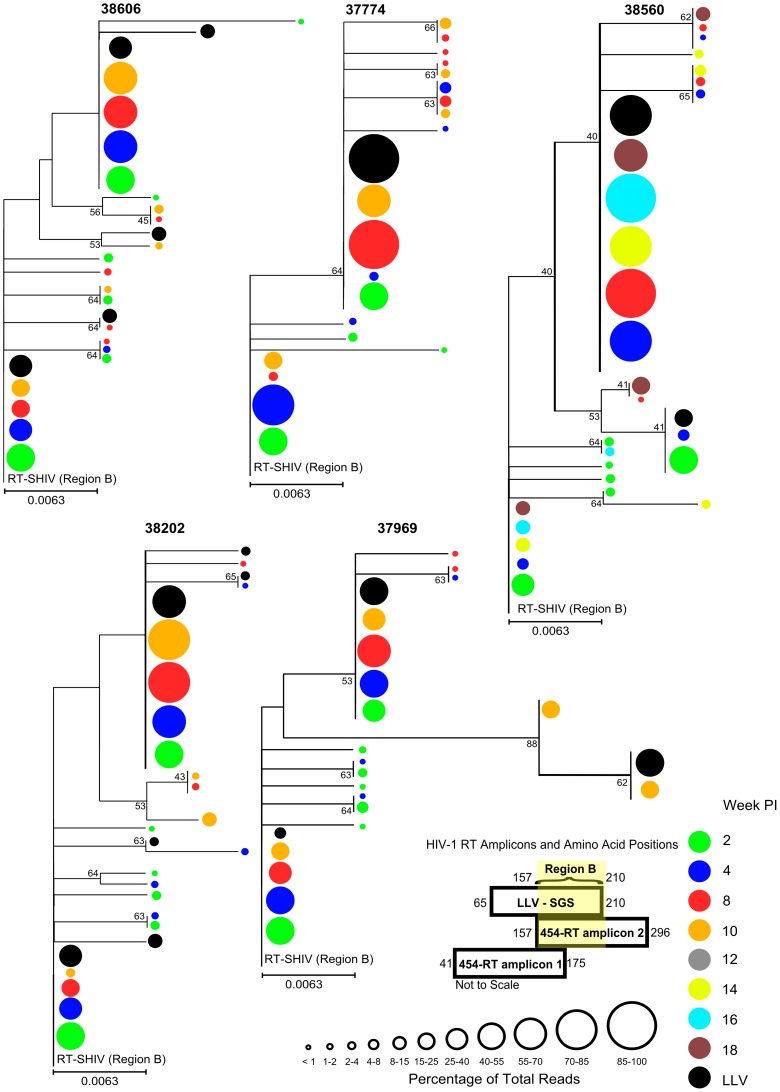
Comparative phylogenetic analysis of pre-suppression and low-level viremia (RT amino acids 157–210). Multiple sequence alignments were performed using 454-RT amplicon 2 pre-suppression 454 consensus sequences and SGA LLV sequences. There were a total of 160 nucleotide positions in the alignment encoding RT amino acids 157 to 210 (region B). Phylograms were constructed and are depicted as described in Figure 2.

Low-level viremia divergence was assessed relative to the pre-suppression profile using a neighbor-joining phylogenetic analysis. The regions sequenced to characterize LLV corresponded to the 3′ end of 454-RT amplicon 1 and the 5′ end of 454-RT amplicon 2. Therefore, in order to compare LLV divergence and allele frequency to pre-suppression viremia, we generated two separate alignments termed region A and region B. As illustrated in Figures 2 and 3 respectively, pre-suppression 454 sequence data for region A was derived from 454-RT amplicon 1 encoding RT amino acids 65 to 175 and pre-suppression sequence data for region B was derived from 454-RT amplicon 2 encoding RT amino acids 157 to 210. Neighbor-joining phylogenetic analyses, similar to those reported by Buzon *et al*., (2011) [43], were performed for each alignment and results are shown in Figures 2 and 3. As determined by visualization of LLV sequence branch lengths relative to those generated from the pre-suppression profile, it is evident that the level of LLV divergence was equivalent to that of the pre-suppression profile. Presumably, this suggests that LLV originated from a reservoir containing archival variant sequences. Only one LLV sequence obtained from RM Mmu 38606 had a divergence greater than the pre-suppression profile (Figure 2; asterisk). However, due to the nonsense mutation observed at amino acid position 88 in RT, this sequence may have been derived from a replication incompetent virion produced from a cell with a replication incompetent proviral sequence. Further analysis indicated that the enhanced divergence observed in this sequence was predominantly due to three G to A mutations, G263A, G412A, and G428A (Table 1; underlined and Figure 2; asterisk). The Los Alamos Hypermut 2.0 program determined that all of these mutations were at 3 of 51 potential ABOBEC3G mutation sites and none of the 36 control sites. The nucleotide and amino acid positions of RT mutations identified within LLV are shown in Table 1. This table reports the frequency that LLV mutations were observed in pre-suppression sequence reads. As was indicated in the phylograms depicted Figures 2 and 3, these data demonstrate that mutations commonly observed in LLV were also regularly observed in the pre-suppression profile.

### Identification of a predominant plasma clone sequence

**Figure 4:**
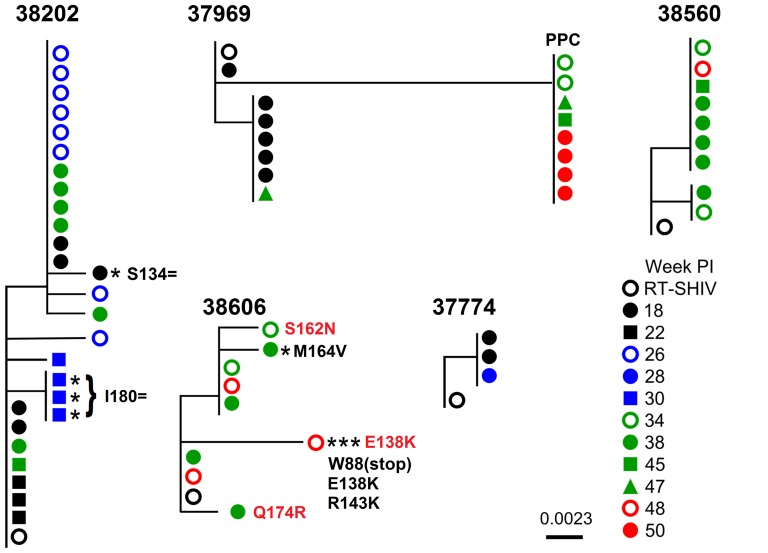
Phylogenetic diversity of low-level viremia. For each RM, phylograms represent RT-SHIV LLV sequences that were obtained between weeks 18 and 50 PI. Sequences were derived by SGA using plasma vRNA samples that were collected after maximal viral load suppression (<50 vRNA copies/mL) was initially achieved. There were a total of 437 nucleotide positions in the alignment encoding reverse transcriptase amino acids 65 to 210. Neighbor-joining phylograms were constructed as described in Figure 2 and were rooted on the consensus sequence of the RT-SHIV inoculum (open black circle). Each asterisk denotes that the indicated LLV sequence contained a single mutation which was not observed in the pre-suppression variant profile. The identity of these mutations has been annotated on the phylograms in black lettering. Drug resistance mutations to compounds which were not used in this study are annotated in red lettering to the right of LLV sequence in which they were observed. These mutations are associated with resistance to the NNRTI Nevirapine (S162N and Q174R) and the NNRTIs Etravirine and Rilpivirine (E138K). Finally, sequences pertaining to the putative predominant plasma clone sequence identified in RM Mmu 37969 have been annotated “PPC”. These sequences were observed at four separate time points (weeks 34, 45, 47, and 50 PI).

A neighbor-joining phylogenetic analysis of full LLV sequences encoding RT amino acids 65–210 is shown in Figure 4. In general, all RMs had limited intra-LLV sequence diversity with sequences clustering into two or three different groups. Limited inter-LLV sequence diversity was also observed in four of the five RMs, with divergence ranging from 0 to 0.007 substitutions/site (0 to 3 mutations/437 nucleotide sites). Interestingly, eight of the 15 LLV sequences obtained during VL suppression from one of the five RMs, Mmu 37969, were markedly more divergent relative to the other RMs (0.023 substitutions/site in Mmu 37969 vs. ≤0.007 substitutions/site in other RMs). Each of these sequences contained the same ten mutations at four different time points during HAART (Figure 4; weeks 34, 45, 47, 50 PI). This finding may indicate the presence of a predominant plasma clone (PPC) sequence. The PPC sequence was not observed before HAART (weeks 2, 4, 8 PI) but was observed two weeks post HAART at week 10 PI (Table 1). For RM Mmu 37969, week 10 PI (VL: 2.6×10^2^ vRNA copies/mL) was also the last time point in the pre-suppression profile (Table 1).

### Presence of low-level viremia mutations not observed in pre-suppression viremia

One criteria for assessing whether or not residual replication occurs during HAART is the evolution of viral sequences during therapy [31]. In comparison to mutations observed in the pre-suppression plasma vRNA profile, two of the five RMs (Mmu 38606 and Mmu 38202) had at least one sequence that contained a mutation which was not previously observed in this data set (Table 1; underlined). Sequences containing these previously unobserved mutations are denoted in Figure 4 by asterisks and the resultant amino acid changes are annotated in black lettering. Each LLV sequence containing a previously unobserved mutation was derived from a plasma sample with a VL <50 vRNA copies/mL and not a transient blip (Table 2; ^c^). In RM Mmu 38202, previously unobserved mutations were identified in two separate samples. Three identical sequences containing a single silent mutation (I80 = ) were identified at week 30 PI and one of the five sequences obtained at week 18 PI also contained a silent mutation (S134 = ). In RM Mmu 38606, two of the nine LLV sequences contained previously unobserved mutations at two separate time points (Figure 4; weeks 38 and 48 PI). These were M164V at week 38 PI, and one sequence from week 48 contained three mutations, W88(stop), E138K, and R143K (Figure 4). For both RMs, none of these previously unobserved mutations were a component of a progressively evolving sequence variant or were resampled at later time points during HAART (Figure 4).

### Characterization of drug resistance mutations in low-level viremia and viral rebound

The region encoding RT amino acids 65 to 210 was sequenced after single-genome amplification of cDNA derived from LLV vRNA. Plasma samples were obtained longitudinally during HAART after maximal VL suppression was achieved. Sequences were compared to the Stanford HIV drug resistance database [44], [45] (accessed March, 27 2013) and no known resistance mutations were identified to drugs used in this study. However, minor drug resistance mutations to the NNRTI Nevirapine (S162N and Q174R) and the NNRTIs Etravirine and Rilpivirine (E138K) were observed in one RM (Mmu 38606). These mutations are annotated in red-lettering next to the corresponding LLV sequence branch shown in Figure 4 (Mmu 38606). Each of these mutations was on a separate LLV sequence, and none were detected at later time points or in more than one sequence. Additionally, two of these mutations S162N, and Q174R were detected at low levels (<2% of reads) in plasma before the initiation of HAART (Table 1; Mmu 38606). These LLV sequences did not assay the presence of known drug resistance mutations to AZT (M41L, T215YF, and K219QE) and Efavirenz (P225H, M230L). Using vRNA extracted from plasma samples collected during initial and peak viral rebound, the region encoding RT amino acids 41 to 296 was sequenced using a 454 sequencer (sequencing depth: 1,000X – 2,000X). Drug resistance mutations to the HAART regimen used were not observed in these samples (data not shown). The Q174R mutation (Nevirapine) was detected in RM Mmu 37774 during peak viral rebound (week 53 PI; 4.3%) but was also observed before HAART (weeks 4 (1.2%) and 8 (1.3%) PI).

### Analysis of reverse transcriptase mutations with putative selective pressures

An analysis of synonymous and nonsynonymous mutations which have putative selective pressures was performed by analyzing mutations that were present in ≥1 plasma sample and were either ≥5% of sequence reads in at least two RMs, or ≥85% in one RM (Table 3). Both pre-suppression (weeks 2 – 18 PI) and post viral rebound (weeks 52 – 69 PI) plasma samples were used in this analysis. From these samples, the region encoding RT amino acids 41 to 296 was sequenced from plasma vRNA using a 454 sequencer (sequencing depth: 1,000X – 2,000X). Identified mutations that were of particular interest were the RT mutations G196R and K275R which were present in at least two RMs and were observed in some samples at levels greater than 60% of sequence reads. In the context of RT-SHIV_mac239_, this study identified the RT mutations G196R and L214F which have been previously observed by our group [46]. To our knowledge, only L214F has been reported in another study by Shao *et al*. [47], using RT-SHIV_mne_ infected pigtailed macaques. Surprisingly, the V75L/L74V which routinely occurs in both our studies [46] and was reported by Shao *et al*. [47], was not observed in any of the five RMs. Although the selective pressures on RT-G196R mutation were notable, 454 sequencing identified this mutation within the viral inoculum in 5% of the 1,900 sequences. Other mutations in the inoculum were also commonly observed in plasma samples though none were identified in >5% of sequence reads in any tested sample (data not shown).

**Table 3:** RT-SHIV mutations with putative selective pressures^a^.

			% of 454 sequence reads in ≥1 sample			
Gene	Nucleotide Change	Amino Acid Change	#≥5%	#≥20%	#≥60%	#≥85%
RT	G612A	K82 =	C, D			
	A521G	Q174R	C, E			
	G586A	G196R^b^	A, B, C, D, E	A, B, C, D, E	A, B, C, E	A, C, E
	G612A	E204 =	B, C **(A, D)**	B		
	G615A	L205 =	A, C, D, E **(B)**	E	E	E
	A623T	H208L	A, D	D	D	D
	C640T	L214Fb,c	D **(E)**	D	D	D
	A824G	K275R	B, E **(A, C, D)**	B, E	B, E	E
	A888G	T296 =	E	E	E	E

a Capital letters indicate the following RMs: (A: Mmu 38202; B: Mmu 37969; C: Mmu 37774; D: Mmu 38560; E: Mmu 38606). In the category “#≥5%” bold RM designations separated in parentheses specify that the indicated mutation was observed in <5% of sequence reads.

b Observed by our group in a previous study [46].

c Observed in RT-SHIV_mne_ infected pigtail macaques (Shao et al. [47]).

## Discussion

Despite the potent efficacy of HAART on HIV-1 replication, residual LLV is not eliminated and viremia rapidly rebounds upon cessation of HAART. In this study, the level of viral suppression (<50 vRNA copies/mL) and the limited frequency of blips was comparable to HIV-1 infected humans during the first 42 weeks of HAART [4], [6]. Given the duration of HAART, an important caveat to our characterization of LLV is that sampled sequences presumably originated from viral production during the 3^rd^ phase of viral decay [48]. While LLV is also observed in humans during this 3^rd^ phase, relatively lower levels of LLV vRNA have been reported in many individuals during a stable 4^th^ phase of viral decay (decay t_1/2_  =  ∞) following six years of HAART [5].

In this study, we hypothesized that a component of LLV was due to the evolution of drug resistance in RT. In the regions sequenced, an analysis of plasma vRNA RT sequences did not identify any known resistance mutations to drugs used in this study. Three minor NNRTI drug resistance mutations to other antiretrovirals were observed in RM Mmu 38606. Two of these mutations (S162N and Q174R; Nevirapine) were observed before the initiation of HAART (Table 1; Mmu 38606). The third mutation, E138K (Etravirine/Rilipvirine), was due to the mutation G412A which is located within an APOBEC3G mutation site. This mutation was linked on a sequence containing two other G to A mutations at similar sites (Figure 4). These mutations were not observed at more than one time point, suggesting that they did not contribute to a population of productively infected cells which persistently contributed to LLV. Despite these findings, it should be considered that the number of LLV sequences obtained from each RM may have been insufficient to detect the remittance of drug resistant variants into the plasma which evolved during HAART.

The regimen used in this study consisted entirely of RT inhibitors, and the sequenced regions encompassed the majority of previously identified RT resistance mutations [49]. Sites encoding drug resistance mutations that were not sequenced in LLV were sequenced during initial and peak viral rebound. In these plasma samples, resistance mutations to the HAART regimen were not observed (data not shown). However, these analyses may be limited due to the fact that low-frequency drug resistance mutations that were selected for during HAART either did not contribute to viral rebound, were below our assay detection sensitivity, or reverted to wild-type sequences during rebound. Nevertheless, as suggested by other human studies [50]–[52], we believe that the selection of drug resistance mutations does not significantly contribute to virions within LLV during maximal VL suppression. This finding does not discount the possibility that selected drug resistance variants may not have been remitted into the plasma due to anatomical barriers or that sufficiently limited residual replication did not result in the marked selection of drug resistant variants in the time frame of this study.

In all five RMs, LLV was characterized by limited intra-sequence diversity which can suggest that a restricted number of sites contribute to LLV. In addition to limited intra-sequence diversity, there was also limited inter-sequence diversity (≤0.007 mutations/site) in LLV sequences obtained from four of the five RMs (Figure 4; Mmu 37774, Mmu 38560, Mmu 38202, and Mmu 38606). The level of divergence and allele diversity was also noticeably similar to the pre-suppression plasma vRNA population (Figures 2 and 3). Collectively, this suggests that limited diversification occurred before the initiation of HAART at eight weeks PI and that sequences within viral reservoirs that contributed to LLV may be relatively homogeneous. Consequently, further insights into the number of sites contributing to LLV are limited given the level of diversification that was achieved in this study.

Whether or not residual replication occurs remains controversial [20]. However, recent observations have shown that HAART intensification with integrase inhibitors can increase the number of 2-LTR circles [53], [54], suggesting that current HAART alone may not effectively penetrate all tissues in some patients. One phylogenetic criteria for assessing whether or not residual replication occurs is the evolution of viral sequences during treatment [31]. Although, the early initiation of HAART limited viral diversification and impeded an analysis of the number of sites contributing to LLV, constrained viral evolution may have enhanced our characterization of baseline diversity. This may have provided a useful tool for assessing viral evolution during HAART. There is yet to be a consensus as to which nucleic acid marker is most suitable in assays to detect viral evolution during therapy. Studies have assessed the evolution of integrated and episomal vDNA sequences in peripheral blood mononuclear cells (PBMC) [43], [55], plasma vRNA [17], [23], cerebral spinal fluid vRNA [17], and vDNA sequences from tissue biopsies [56], [57]. During HAART, we chose to study plasma vRNA due to previous findings that a genetic linkage between PBMC proviral DNA sequences and either LLV [17], [18], [58] or episomal DNA sequences in PBMC [43] is often not well defined. This indicates that LLV may originate from sources other than infected PBMC such as tissue viral reservoirs. In addition to the lack of a defined marker, inconclusive findings concerning residual replication may be due to the difficulty in sampling sites of viral persistence and establishing a baseline of diversity before VL suppression.

After maximal VL suppression, we sampled LLV sequences in two of the five RMs (Mmu 38202 and Mmu 38606) that contained mutations which were not previously observed in our analysis of viral diversity in pre-suppression viremia (Figure 4; asterisks). These findings may suggest that residual replication occurred during HAART. However, these mutations were not a component of a temporally evolving sequence or re-sampled at later time points. This may indicate that the level of residual replication was insufficient to efficiently remit these virions into the plasma at later times or that the level of sampling was insufficient to detect them. On the other hand, it should be considered that these variants emerged before VL suppression yet were either not remitted into pre-suppression plasma or not detected in our analysis of the pre-suppression variant profile.

The use of high-throughput sequencing to characterize baseline diversity provided an enhanced ability to detect mutations relative to more traditional SGA and PCR cloning strategies of pre-suppression viremia. For example, 454 sequencing correctly identified several LLV mutations which existed before VL suppression and were presumably not the result of residual replication. A few of these LLV mutations include RT-G612A in RM Mmu 38202 and RT-A521G in RM Mmu 38606 which were observed in ≤1% of sequences (Table 1). However, this assay was not able to detect all of the low-frequency (<2% of reads) mutations present in two matched plasma samples. Thus, as an alternative to assessing LLV allele prevalence relative to the pre-suppression profile, we also performed a comparative analysis of LLV divergence (Figures 2 and 3). This was performed to investigate the hypothesis that LLV variants that were the product of residual replication may be more divergent than variants which evolved before VL suppression. These analyses demonstrated that LLV had an equivalent level of divergence to pre-suppression viremia (Figures 2 and 3) and was also frequently marked by alleles that were commonly observed in the pre-suppression profile (Table 1). Similar to well suppressed humans [17], [18], [59] and the previous study by Kearney *et al*., in pigtailed macaques [31], our findings suggest that a stable viral reservoir containing archival variant sequences, such as the latent viral reservoir, principally contributed to LLV.

Phylogenetic analyses of residual viremia in humans have shown that LLV in many individuals is frequently characterized by the presence of oligoclonal populations of viral sequences which have been termed predominant plasma clones (PPC) [17], [18], [25]–[27]. In this study, it is possible that one RM (Mmu 37969) maintained a PPC sequence during therapy for a period of at least 16 weeks. To our knowledge, this is the first report to suggest that this enigmatic feature of human LLV is mimicked in an animal HAART model. Interestingly, the majority of PPC sequence mutations were not identified before HAART, but were identified two weeks after therapy at week 10 PI (Table 1; Mmu 37969) following a reduction in viral loads from 10^5^ to 10^2^ vRNA copies/mL (Figure 1). While this sequence may have arisen due to viral evolution in the two weeks after initiation of therapy, we hypothesize that a reservoir remitting virions containing the PPC sequence already existed at low levels before therapy. Following initiation of HAART, we were able to detect this pre-existing PPC sequence after the “noise” of ongoing viral production from other sites was reduced. It has been hypothesized that a source of chronically infected cells contributes to LLV in some patients [59]. A population of chronically infected cells remitting virions with PPC sequences would not be sensitive to the addition of RT inhibitors because the mechanism of action acts up stream of the viral replication cycle. Moreover, viral production from these cells may be sustainable due to reduced viral induced cytopathic effects. Putative evidence for this hypothesis has been provided by the apparent lack of viral evolution in PPC sequences during HAART [17], [25], [59] and studies demonstrating that LLV vRNA levels are often not affected by HAART intensification [53], [59], [60]. Alternatively, because some viral reservoirs are maintained by homeostatic proliferation [61], it has been hypothesized that a self-renewing latent viral reservoir contributes to PPC viral populations [25]. Despite these findings, the mechanisms contributing to PPC viral populations remain undefined.

Similar to humans [17], [18], [25]–[27], the absence of PPC sequence evolution in RM Mmu 37969 suggests that residual replication did not occur following PPC viral production and that these populations may not be maintained via residual replication. Surprisingly, the PPC sequence was not observed at week 18 PI (Figure 4) and sequences were not generated at week 28 PI (Table 2). This finding may suggest that there are natural fluctuations in viral production from PPC-infected cells or that these cells are located in sites with barriers to viral remittance into the plasma. It is conceivable that LLV sequences from other RMs were also a result of the uncharacterized circumstances that produce PPC viral populations in humans (Figure 4). However, in these RMs, it was not possible to identify virions emerging from putative PPC viral populations given the low level of LLV divergence relative to the pre-suppression profile (Figures 2 and 3). It may have been serendipitous that the PPC sequence emerged in a viral population with a marked level of divergence relative to the limited level of diversification observed in other RMs. Thus to investigate this phenomenon further, it may be advantageous to allow further viral diversification to occur before initiating therapy or to utilize a more heterogeneous viral inoculum. Additionally, a long term study is warranted in order to further support our hypothesis that the enigmatic circumstances that give rise to PPC viral populations in humans are mimicked in this model.

Similar to many human studies [17], [18], [25], data generated in this study suggests that there was an apparent lack of viral evolution in LLV. However, we cannot exclude the possibility that instances of residual replication did occur within these RMs in cryptic anatomical reservoirs. An ideal non-human primate model of HIV-1 HAART can play an important role in future attempts to eliminate residual LLV, control persistent immune activation, and pursue viral eradication strategies [62]. In conjunction with our previous quantitative analyses of anatomical viral reservoirs [32] and LLV [48] we believe that this study further supports the utility and relevance of an RT-SHIV rhesus macaque HAART model in studies designed to improve human health and eradicate HIV-1 infection.

## Materials and Methods

### Ethics statement

The use of rhesus macaques (Macaca mulatta) in this study was approved by the Association for the Assessment and Accreditation of Laboratory Animal Care, International (AAALAC) accredited University of California, Davis Institutional Care and Use Committee (IACUC), animal use assurance number (A-3433-01). The UC Davis IACUC has an Animal Welfare Assurance on file with the Office of Laboratory Animal Welfare (OLAW). Rhesus macaques were administered 10 mg/kg body weight ketamine-HCl (Parke-Davis, Morris Plains, NJ, USA) intramuscularly when necessary for immobilization. At necropsy, macaques were sedated with ketamine-HCl and then humanely euthanized with a barbiturate overdose. Additionally, analgesics were administered at the discretion of the CNPRC veterinary staff in an effort to minimize all animal pain and discomfort. Macaques were housed at the California National Primate Research Center (CNPRC), which is fully accredited by the Association for the Assessment and Accreditation of Laboratory Animal Care (AAALAC). For housing, macaques were maintained in cages with 4 square feet of floor space, or 6 square feet if over 10 kg, and fixed perch bars in a temperature controlled BSL-2+ vivarium with continuous monitoring of temperature and humidity. Compatible macaques were paired continuously or intermittently (separated at night) whenever possible. All macaques had visual and auditory access to other macaques 24 hours per day. These macaques were fed a balanced commercial macaque chow (Purina Mills, Gray Summit, MO) twice daily, fresh produce twice weekly, and had free access to water 24 hours per day. Supplemental food was provided when clinically indicated. Environmental enrichment was provided daily, included manipulanda (forage boards, mirrors, puzzle feeders) and novel foodstuffs. The endpoint of this study was set at a pre-specified time point as part of the experimental design of the antiretroviral treatment regimen. All macaques were humanely euthanized by overdose of sodium pentobarbital (60 mg/kg) administered by the intravenous route under ketamine sedation (10 mg/kg).

### Virus and rhesus macaques

The RT-SHIV inoculum was prepared by transfecting CEMx174 cells with the 5′ and 3′ RT-SHIV plasma half clones and subsequently collecting the virus from cell culture supernatants as previously described [40]. The Feline Leukemia retrovirus (FeLV) was used to facilitate RT-SHIV recovery during ultracentrifugation. FeLV was collected and stored from tissue cultures of the chronically infected feline lymphoblastoid cell line FL74 [63] as previously described [48].

A total of five, adolescent female rhesus macaques (RMs) of mixed Chinese-Indian ancestry were used in this study. Each RM was from the retrovirus-free colony of the CNPRC and weighed 3 to 5 kg during the course of the study. Infections were synchronized by intravenously infecting all RMs with 1×10^5^ 50% tissue culture infectious doses (TCID_50_) of RT-SHIV. Eight weeks PI, a HAART regimen consisting of five antiretroviral agents was initiated for all five RMs. Rhesus macaques Mmu 38202 and Mmu 37969 were necropsied during treatment after 42 weeks of continuous therapy. Therapy was intentionally discontinued at week 50 PI for the remaining three RMs: Mmu 38560, Mmu 38606, and Mmu 37774. These RMs were necropsied between weeks 65 and 69 PI.

### Preparation and administration of drugs

As in our previous studies [32], [40], RMs were treated with a frontline antiretroviral regimen consisting of tenofovir (PMPA), emtricitabine ((-)-FTC), and efavirenz (EFV; Sustiva) [1]. In this study, zidovudine (AZT) and amdoxovir (DAPD) were also used. Stock solutions of (-)-FTC, and PMPA were prepared as previously described [48]. The nucleoside RT inhibitors (-)-FTC, PMPA, and AZT were administered subcutaneously at 16 mg/kg QD, 30 mg/kg QD, and 30 mg/kg BID respectively. Subcutaneous drug administration improves compliance and these drugs are sufficiently water soluble to be administered in this fashion. PMPA [64], AZT [65] and (-)-FTC [66] have high bioavailability when administered subcutaneously and penetrate RM tissues at levels comparable to humans [64]–[66] . However, one recent study of RM gastrointestinal tissues has reported that subcutaneous delivery of PMPA (Tenofovir) may result in a lower drug concentration than oral delivery of the pro-drug Tenofovir disoproxil fumarate which is used in humans [64]. This finding may represent an important caveat to the level of drug delivery and the potential effects on residual replication in humans versus non-human primates which receive some drugs subcutaneously. Both EFV and DAPD were administered orally in a variety of edible foods at 200 mg per day and 85 mg per day respectively. These drugs were first compounded into a paste using a 65% sucrose solution at a defined mass of drug/cc^3^ of paste (additional details are available upon request). To offset the bitterness of these drugs, a precise quantity of the “drug-paste” was vigorously blended into either peanut butter or strawberry/apricot jam which was then applied to food treats. To enhance compliance, all RMs received their daily oral EFV and DAPD doses prior to receiving their main meal and were monitored to ensure that they completely consumed the drug laced food. Drug dosages were adjusted weekly according to body weight. To reduce adverse side effects, the dose of PMPA and AZT was reduced at week 15 PI to 15 mg/kg and 10 mg/kg respectively for all RMs. For RM Mmu 37774, the dose of PMPA was further reduced to 7.5 mg/kg (week 31 PI) and subsequently to 5 mg/kg (week 35 PI). For RM Mmu 38560, the dose of PMPA was also further reduced to 7.5 mg/kg at week 40 PI.

### Isolation and quantitation of plasma viral RNA

EDTA-anticoagulated blood samples (10 to 15 mL) were collected both before HAART and during therapy at approximately two week intervals. After cessation of therapy, samples were obtained weekly for at least five weeks. Cell-free plasma was obtained after centrifugation of whole blood and archived at −80°C until vRNA extraction. RT-SHIV vRNA was measured longitudinally in order to select samples for sequence analysis. Viral RNA was extracted as previously described [67] from 140 µL of plasma using a commercial viral RNA mini kit (Qiagen, Valencia, CA, USA). All qRT-PCR assays were performed using either an ABI 7900 or 7500 thermal cycler (Applied Biosystems, Foster City, CA). Each reaction contained 10 µL RNA template, 1X AmpliTaq Gold Buffer A, 5 mM MgCl_2_, 0.8 µM dNTP, 1.2 µM of each forward and reverse primer, 40 nM probe, 0.1 U/µL AmpliTaq Gold, and 0.02 U/µL MuLV RT (Applied Biosystems). Plasma vRNA that was sequenced in order to characterize the pre-suppression profile was isolated according to the vRNA extraction component of the ultracentrifugation virus load assay described in 2010 by Deere *et al*. [48]. This procedure was originally adapted from the vRNA isolation procedure described by Palmer *et al*. [3] that is commonly used in the HIV-1 single copy assay. This protocol was modified from our previously published assay [48] to include 7 units/sample of proteinase K from a buffered aqueous glycerol solution instead of 200 µg/sample of lyophilized powder. Seven units of aqueous proteinase K represented the average number of bioactive units in 200 µg of lyophilized powder. This change had no effect on assay sensitivity (data not shown). Isolated vRNA was resuspended in 45 µL of nuclease free water.

Half of the vRNA obtained from the pre-suppression plasma samples was quantified by the previously described SIVgag qRT-PCR assay. It was essential to preserve a significant quantity of the isolated vRNA for 454 sequencing, thus only duplicate reactions were performed and the volume of template vRNA was reduced from 10 to 7 µL. In addition, a single control reaction in the absence of RT was used to estimate the contribution of vDNA sequences. In order to assess vRNA recovery, FeLV was quantified by the previously described qRT-PCR protocol [48]. This protocol was modified to reduce the volume of vRNA template from 5 to 2 µL.

To determine the genetic diversity of LLV, after maximal VL suppression was initially achieved (<50 vRNA copies/mL), vRNA in available plasma samples was isolated by the previously described ultracentrifugation virus load assay vRNA extraction procedure (see above). Extracted LLV vRNA was resuspended in 21.4 µL of nuclease free water.

PCR amplification and 454 pyrosequencing

Pre-suppression plasma vRNA was converted to cDNA using AccuScript High Fidelity Reverse Transcriptase (Agilent Technologies, Santa Clara, CA, USA). For each sample, a 40 µL, random 9-mer primed cDNA synthesis reaction containing 20 µL of vRNA template was prepared according to the manufacturer's instructions. Reaction conditions were amended to use a 90 minute 42°C elongation step. For each sample, we targeted two approximately 450 nucleotide regions within RT encoding amino acids 41 to 296 (Table S1; 454-RT amplicon 1 and 454-RT amplicon 2). Amplicons were generated by the universal tag method [68] using custom universal tag sequences TagF and TagR (Table S1). All conventional PCR was performed using Phusion high-fidelity DNA polymerase (New England Biolabs (NEB), Ipswich, MA, USA) and a Mutltigene Gradient Thermal Cycler (Labnet, Woodbridge, NJ, USA). The reaction and thermal cycling conditions for conventional PCR procedures are described in Table S2. First round 454 target amplification (Table S2; 454-Round 1) was performed in a 50 µL final reaction volume containing 4 µL of cDNA template. Reports by Shao *et al.* [69] and others [70] demonstrated that PCR induced recombination can be significantly reduced by increasing PCR elongation times. Consequently, elongation times were approximately three times greater per base than those recommended by the manufacturer. Addition of 454-titanium adaptor sequences and multiplex identifiers was performed in a second 30 µL PCR reaction (Table S2; 454-Round 2) using 0.5 µL of 454-Round 1 PCR product. Previous studies have demonstrated that over amplification by PCR increases both the number of mutations [71] and the level of PCR induced recombination [72]. Consequently, second round PCR was performed using a gradient of amplification cycles in order to amplify each region with the fewest number of cycles required to visualize the amplicon by ethidium bromide staining. It was empirically determined that the number of cycles (Y) required was dependent on the original vRNA concentration (X) (copies/µL) of the resuspended vRNA. The equation Y  =  −1.98ln(X) + 35 was predictive of the required cycle number and was used for all samples that had VLs between 1×10^7^ and 5×10^3^ vRNA copies/mL. This equation was not suitable for samples with VLs <5×10^3^ vRNA copies/mL. These samples were amplified for 30, 35, and 40 cycles and the reaction that generated a visible band with the fewest number of cycles was sequenced.

After electrophoresis through a 1.5% agarose gel, ethidium bromide stained amplicons were visualized using a Dark Reader transilluminator (Clare Chemical Dolores, CO, USA) and the correctly sized band was excised and purified using the NucleoSpin Gel and PCR Clean-up System (Macherey Nagel, Bethlehem, PA, USA). Using an Agilent Bioanalzyer DNA7500 chip, preliminary analysis of amplicons generated in this study indicated that gel extracted amplicons were not contaminated by detectable levels of primer-dimers. Quantitation was performed by fluorometry using the Quant-iT PicoGreen dsDNA Assay Kit (Invitrogen, Carlsbad, CA, USA). Amplicons were diluted in 5 mM Tris-HCl pH 8.0 to 2×10^8^ molecules/µL and then pooled in equal molar concentrations to create sequencing libraries. Emulsion PCR (emPCR) was performed using the titanium library-A emPCR reagents (Roche/454 Branford, CT, USA) according to the manufacture's long-fragment reaction and cycling conditions that are described in technical bulletin No. 2011-011 “Amplicon Sequencing with Various emPCR Amplification Conditions”. Library titration analysis determined that an input ratio of 0.6 molecules per bead would result in an average enrichment of 5 to 10%. Bi-directional 454-pyrosequencing was performed using a GS Junior Sequencer (versions 2.5p1 - 2.7). Sequencing results were analyzed by the 454 GS Run Processor version 2.7 using the default amplicon signal processing pipeline.

### Single-genome amplification of LLV samples

Viral RNA was isolated from plasma samples obtained after initial maximal VL suppression was achieved (<50 vRNA copies/mL), according to the previously described ultracentrifugation based vRNA extraction procedure (See above). Viral RNA was converted to cDNA using AccuScript RT (Agilent, Santa Clara CA) in a gene-specific primed cDNA synthesis reaction. The previously described reaction conditions were modified to use 21.4 µL of vRNA template, 2 µM of the gene-specific primer RT-SGA-GSP (Table S1), and omitted the initial 25°C elongation step. After cDNA synthesis, 10 U of RNAse H (NEB) and 4.4 µL 10X RNaseH buffer was added to the reaction mixture which was then incubated at 37°C for 20 min. The first round of PCR (Table S2; SGA-RT-Round 1) was performed by preparing 16, 25 µL reactions containing 2.5 µL of cDNA per reaction. A nested PCR amplification step (Table S2; SGA-RT-Round 2) was performed in a 20 µL reaction volume containing 0.5 µL of 1^st^ round PCR product. PCR reactions were screened by agarose gel electrophoresis. Positive amplification products were reamplified in a 50 µL reaction volume, purified, and bi-directionally sequenced using primers RT-SGASeqF and RT-SGASeqR (Table S1) by the University of Michigan's DNA sequencing core according to protocols for Applied Biosystems DNA Sequencers (Model 3730 XL).

### Phylogenetic analysis

To generate the population of unique viral sequences within each sample, single 454 sequence reads were condensed if the sequences were determined to be likely identical by the Roche Amplicon Variant Analysis software version 2.7. This procedure generated a single consensus sequence which was paired with a known quantity of individual reads from which it was derived. Due to the high insertion and deletion error rate in homopolymer regions, insertion and deletion mutations within homopolymer regions (n≥2) were manually reverted to wild type and then re-merged with identical variants. This was accomplished in MEGA 5.2 [73] following a multiple sequence alignment of 454 consensus sequences using MUSCLE [74]

Because the LLV SGA data regions did not directly correspond to the pre-suppression 454 sequences, a phylogenetic analysis of viral divergence was performed after first generating two separate alignments termed region A and region B. As illustrated in Figure 2, the 333 nucleotide region A alignment was created by aligning the region encoding RT amino acids 65 to 175 of the 454-RT amplicon 1 (Table S1) pre-suppression sequences to the corresponding LLV sequence region. Similarly, the 160 nucleotide region B alignment was created by aligning the region encoding RT amino acids 157 to 210 of the 454-RT amplicon 2 (Table S1) pre-suppression sequences to the to the corresponding LLV sequence region (Figure 3). All positions containing gaps and missing data were eliminated and the evolutionary history was inferred using the Neighbor-Joining method [75]. Phylograms were drawn to scale, with branch lengths in the same units as those of the evolutionary distances used to infer the phylogenetic tree. The evolutionary distances were computed using the Tamura-Nei method [76] and are in the units of the number of base substitutions per site. This method was selected using the Model Selection tool implemented in MEGA 5.2 [73] which was predicted to be the most accurate model for all alignments generated in this study. Phylograms were rooted on the consequence sequence of the RT-SHIV inoculum which, as expected, was determined to be the most recent common ancestor sequence from each RM.

### Mutation and statistical analysis

The consensus sequence of the RT-SHIV inoculum was used to generate a reference sequence for all alignments in this study. Roche's GS Amplicon Variant Analysis software version 2.7 was used to de-multiplex, align, and detect putative mutations in standard flow-gram formatted 454-sequence data. For each amplicon, the differential abundance of specific mutations in complementary read orientations was compared. This analysis was used to remove the majority of mutations that were more than twice as abundant in one of the read orientations. Exceptions were made for mutations that were up to five times more abundant in one read orientation in circumstances where this discrepancy was likely explained by an equivalent bias in the number of reads generated in each orientation. Due to the high 454 sequencing error rate in homopolymer regions, insertion and deletion mutations were not considered. Finally, all substitution mutations that were observed in more than 0.5% of sequence reads and also met the previously described criteria were selected and further analyzed in Microsoft Office Excel 2007.

APOBEC3G mutation site analysis was determined using the Los Alamos National Laboratory HIV sequence analysis tool Hypermut 2.0 [77].

Statistical analyses described in the text were performed using GraphPad Prism version 6.01 for Windows, GraphPad Software (San Diego, CA, USA).

### Access to sequence data

Reverse transcriptase 454 consensus sequence alignments used in this study that were generated from the RT-SHIV inoculum, pre-suppression, rebound, and necropsy plasma samples are available in a supplementary Microsoft Excel Spreadsheet (Sequences S1). For each time point there are two separate alignments representing amplicon 1 (encoding RT amino acids 41–175) and amplicon 2 (encoding RT amino acids 157–296). Also provided in this spreadsheet are alignments of single-genome sequence data (encoding RT amino acids 65–210) generated from RM Mmu 37969 week 10 PI, and all LLV samples that were successfully amplified. This report also describes the number of sequences generated for each amplicon. Additional sequence information is available upon request.

## Supporting Information

Sequences S1 **Sequences used in study.** Reverse transcriptase 454 consensus sequence alignments used in this study that were generated from the RT-SHIV inoculum, pre-suppression, rebound, and necropsy plasma samples are available in a supplementary Microsoft Excel Spreadsheet. These data are separated by individual tabs within the spreadsheet. For each time point there are two separate alignments representing amplicon 1 (encoding RT amino acids 41–175) and amplicon 2 (encoding RT amino acids 157–296). Also provided in this spreadsheet are alignments of single-genome sequence data (encoding RT amino acids 65–210) generated from RM Mmu 37969 week 10 PI, and all LLV samples that were successfully amplified. This spreadsheet also describes the number of sequences generated for each amplicon.
(XLSX)

Table S1 **Primer sequences.**
(DOCX)

Table S2 **Reaction and cycling conditions for conventional PCR reactions.**
(DOCX)
