# Supplementary material for: Residual Viremia in an RT-SHIV Rhesus Macaque HAART Model Marked by the Presence of a Predominant Plasma Clone and a Lack of Viral Evolution
Source: PLoS One. 2014 Feb 5;9(2):e88258. doi: 10.1371/journal.pone.0088258 (PMC3914964; doi:10.1371/journal.pone.0088258)
Supplement: Table S1 — Primer sequences. (DOCX) [file pone.0088258.s002.docx]

**Table S1.** Primer sequences

| Amplicon ID | **Amino acids Amplified** | **Primer ID** | **Primer Sequence 5’ – 3’** |
| --- | --- | --- | --- |
| SGA – Target Amplification | | | |
|  | 233 to 226**^a^** | RT-SGA-GSP | GTTCATAACCCATCCAAAGGAATG |
| SGA-RT-Round 1 | 59 to 215**^b^** | RT-SGA-Round 1F | GGCCTGAAAATCCATACAATAC |
|  |  | RT-SGA-Round 1R | CTGATGTTTTTTGTCTGGTGTG |
| SGA-RT-Round 2 | 65 to 210**^b^** | RT-SGA-Round 2F | TACTCCAGTATTTGCCATAAAG |
|  |  | RT-SGA-Round 2R | GTGGTAAGTCCCCACCTC |
| SGA-Sequencing Primers | | | |
|  | 86 to 93**^a^** | RT-SGASeq-F | CTTCTGGGAAGTTCAATTAGGA |
|  | 197 to 190**^a^** | RT-SGASeq-R | GCTGCCCTATTTCTAAGTCAGAT |
| Universal Amplicon Tags | | | |
|  | - | TagF | CGGAACTCACTGCTCATACC |
|  | - | TagR | CAGTCCAGCTACGCTGACTC |
| 454-Target Amplification | | | |
| 454-RT amplicon 1 | 41 to 175**^b^** | RT-454 (1) F | TagF-AAGCATTAGTAGAAATTTGTACAGAG |
|  |  | RT-454 (1) R | TagR-CATGTATTGATAGATAACTATGTCTGG |
| 454-RT amplicon 2 | 157 to 296**^b^** | RT-454 (2) F | TagF-ACAGGGATGGAAAGGATCAC |
|  |  | RT-454 (2) R | TagR-GCCAGTTCTAGCTCTGCTTCTTC |
| 454 – Barcoded Fusion Primers | | | |
|  | - | Universal F | Titanium(A)-MID#-TagF |
|  | - | Universal R | Titanium(B)-MID#-TagR |
|  | - | Titanium (A) | CGTATCGCCTCCCTCGCGCCATCAG |
|  | - | Titanium (B) | CTATGCGCCTTGCCAGCCCGCTCAG |
| 454- Multiplex Identifiers (MID) | | | |
| MID 1 | ACGAGTGCGT | MID 10 | TCTCTATGCG |
| MID 2 | ACGCTCGACA | MID 11 | TGATACGTCT |
| MID 3 | AGACGCACTC | MID 13 | CATAGTAGTG |
| MID 4 | AGCACTGTAG | MID 15 | ATACGACGTA |
| MID 5 | ATCAGACACG | MID 16 | TCACGTACTA |
| MID 6 | ATATCGCGAG | MID 19 | TGTACTACTC |
| MID 8 | CTCGCGTGTC |  |  |

^a^For sequencing and gene-specific cDNA synthesis (GSP) primers, the region shown is the approximate region where the primer anneals. This region is denoted in the 5’ to 3’ orientation of the primer. Amino acids are numbered beginning with the first RT amino acid where the primer anneals.

^b^For PCR primer pairs, the region shown depicts the amplified region after trimming PCR primers.
