# Supplementary material for: Residual Viremia in an RT-SHIV Rhesus Macaque HAART Model Marked by the Presence of a Predominant Plasma Clone and a Lack of Viral Evolution
Source: PLoS One. 2014 Feb 5;9(2):e88258. doi: 10.1371/journal.pone.0088258 (PMC3914964; doi:10.1371/journal.pone.0088258)
Supplement: Table S2 — Reaction and cycling conditions for conventional PCR reactions. (DOCX) [file pone.0088258.s003.docx]

**Table S2.** Reaction and cycling conditions for conventional PCR reactions

| Reaction Conditions | | | | | | | | |
| --- | --- | --- | --- | --- | --- | --- | --- | --- |
| PCR Reaction Name | **Primers Used** | **Volume (µL)** | **Template (µL)** | **Primers [nM]^d^** | **Buffer (1X)** | **dNTP [mM]** | **Phusion^a^ (U/µL)** | **MgCl_2_^b^ [mM]** |
| **454-Round 1** | 454 - Target Amplification | 50 | 4 µL cDNA | 1000 | Phusion HF | 0.2 | 0.02 | 2.3 |
| **454-Round 2** | 454 - Barcoded Fusion Primers | 30 | 0.5 µL 454-R1 | 1000 | Phusion HF | 0.2 | 0.02 | 2.3 |
| **SGA-RT-Round 1** | RT-SGA-Round 1 F & R | 25 | ~2.5 µL cDNA | 500 | Phusion HF | 0.2 | 0.02 | 1.5 |
| **SGA-RT-Round 2** | RT-SGA-Round 2 F & R | 20 | 0.5 µL SGA-RT-R1 | 500 | Phusion HF | 0.2 | 0.02 | 1.5 |
| Thermal Cycling Conditions | | | | | | | | |
| PCR Reaction | **Initial Denature** | **Cycles** | **Annealing** | **Elongation** | **Melt** | **Final Extension** | |  |
| **454-Round 1** | 98°C for 75 s | 25 | 61°C for 30 s | 72°C for 48 s | 98°C for 15 s | 72°C for 5 min | |  |
| **454-Round 2** | 98°C for 75 s | Y^c^ | 64°C for 15 s | 72°C for 55 s | 98°C for 15 s | 72°C for 5 min | |  |
| **SGA-RT-Round 1** | 98°C for 55 s | 25 | 62°C for 30 s | 72°C for 30 s | 98°C for 10 s | 72°C for 5 min | |  |
| **SGA-RT-Round 2** | 98°C for 55 s | 35 | 56°C for 15 s | 72°C for 30 s | 98°C for 10 s | 72°C for 5 min | |  |

^a^Phusion High-Fidelity DNA polymerase (New England Biolabs, Ipswich, MA, USA)

^b^MgCl_2_ is present at 1.5 mM in 1X Phusion buffer. Reactions with >1.5 mM MgCl_2_ were supplemented with concentrated MgCl_2_.

^c^Variable number of cycles dependent on estimated target copy number. The equation Y = -1.98ln(X) +35 was found to be highly predictive of the required cycle number and was used for all samples that had viral loads between 1 x 10^7^ and 2 x 10^3^ vRNA copies/mL. This equation was not suitable for samples with viral loads <2 x 10^3^ vRNA copies/mL. These samples were amplified for 30, 35, and 40 cycles and the reaction that generated a visible band with the fewest number of cycles was sequenced.

^d^Each primer is at the specified concentration
